# Hiding scrambled text messages in speech signals using a lightweight hyperchaotic map and conditional LSB mechanism

**DOI:** 10.1371/journal.pone.0296469

**Published:** 2024-01-03

**Authors:** Mustafa A. Al Sibahee, Zaid Ameen Abduljabbar, Chengwen Luo, Jin Zhang, Yijing Huang, Iman Qays Abduljaleel, Junchao Ma, Vincent Omollo Nyangaresi

**Affiliations:** 1 National Engineering Laboratory for Big Data System Computing Technology, Shenzhen University, Shenzhen, PR China; 2 Computer Technology Engineering Department, Iraq University College, Basrah, Iraq; 3 Department of Computer Science, College of Education for Pure Sciences, University of Basrah, Basrah, Iraq; 4 Shenzhen Institute, Huazhong University of Science and Technology, Shenzhen, China; 5 Department of Computer Science, College of Computer Science and Information Technology, University of Basrah, Iraq; 6 College of Big Data and Internet, Shenzhen Technology University, Shenzhen, China; 7 Department of Computer Science and Software Engineering, Jaramogi Oginga Odinga University of Science & Technology, Bondo, Kenya; Military Institute of Science and Technology, BANGLADESH

## Abstract

This study presents a lightweight, secure audio steganography system for hiding text messages for transmission over the Internet, with the aim of addressing the current problems of high computational cost and insufficient security identified in earlier studies. We propose a two-phase functioning mechanism. Text characters are first transformed into ASCII code and stored in a vector, which is then divided into three sub-vectors. These sub-vectors are scrambled using two low-complexity operations, namely a forward-backward reading technique and an odd-even index. Two scrambling loops are performed, the first on the small sub-vectors the second on the vector as a whole. In the hiding phase, the speech signal samples are divided into 256 blocks using only 200 values per block, and low-complexity quadratic and the Hénon maps are used to hide the speech signal in a random manner. The conditional LSB is applied as a low-complexity algorithm to identify hidden bits, and a special hyperchaotic map algorithm is developed to randomly choose locations. The proposed approach provides good security for a scrambled text message, with high SNR and PSNR, small MSE and PESQ, a SSIM value of close to one (As indicated in Tables 1, 2, 3, and 4), a BER value of close to zero (as shown in table 8), NCC value near +1 (as shown in table 8), and an MOS value of near five (as described in table 6), as well as a low computational hiding cost.

## Section 1: Introduction

Over the last decade, communication methods have changed significantly, with digital media now providing the main communication channel. In all walks of business and private life, people are moving away from the use of paper documents and transitioning towards email and other forms of digital media. The technical revolution means that limitless memory and processing capability are now available, and it is now part of everyday life to exchange images, text, audio, and videos over the Internet. However, with this technology comes the requirement to maintain the confidentiality, security, and privacy of the data that are transmitted [1]. Developers of data systems need to carefully consider information security, including confidential data transfer, access control systems for digital content distribution, secret data storage, modification protection, and media database systems [2]. It is often necessary to protect the security of information using encryption technology and information hiding.

Since security requirements are continuing to increase, encryption alone is no longer sufficient, and steganography also needs to be applied to further enhance security and to maintain the integrity and confidentiality of data. When encryption is used, text can only be read by someone who knows the private key, meaning that encrypted text cannot be decrypted without it [3, 4], whereas in steganography, confidential data are hidden from unauthorized individuals using a method that is also known as “covered writing.” Steganography aims to hide the fact that a secret message exists, rather than to conceal its content.

Currently, the most frequent application of steganography is to hide one digital file within another [5]. In general, stego-data hiding requires three distinct characteristics that primarily act at the application level: security, capacity, and resilience. The secret bits contained within a stego-cover or ‘envelope’ are pointed to as their capacity. The concept of security relies on the impossibility of an intruder discovering the hidden secret information, while robustness comes from the capacity to withstand changes, reformatting, or the loss of secret, unseen data [6].

In data hiding, four primary factors are found [7]: 1) Hiding capacity is the maximum size of a concealed secret text.2) Imperceptibility manifests as indistinguishability between the cover and stego sounds. 3) Secure denotes the imperceptibility of the secret communication and its undetectability. 4) Robustness denotes the capacity to effectively recover a secret message with more accuracy, as well as the capacity of a stego file to survive different assaults.

Depending on the type of cover object, steganography technologies can be divided into five types: image, video, network, text, and audio steganography [2, 5]. The hidden data that can be inserted into the cover media depends on the perceptual redundancy of the human organs and the statistical redundancy of the digital medium [8, 9].There has been a great deal of interest in hiding data information in audio signals [10, 11], as these contain significantly more redundant data than other types [12–15]; however, as the human auditory system (HAS) is very sensitive to slight variations in sound signals, hiding data in an audio cover is more difficult than in other types of covers (images, texts) [9, 13, 16–19]. In addition, a minor change in audio data may have a considerable effect on its meaning. Thus, the transmission of hidden audio data represents a difficult task. The audio carrier may transport several types of secret data, including text, audio, and images. Time domain, transfer domain, compressed domain, phase, and echo algorithms have all been used in audio steganography.

Various models and strategies for enabling voice hiding techniques have been proposed in the past few years. Most of the previous works in this area [3, 20–26] suffer from limitations in terms of the time needed to hide the data, the large amount of memory allocation required for embedding and extraction, poor signal-to-noise ratio (SNRs), high bit error rates (BERs), and large mean square error (MSE). Recent schemes are also unsuitable for resource-constrained devices, due to the high computing overheads involved. Moreover, the HAS is very sensitive to sound modification, and these limitations will be discussed further in the next section. A lightweight method is therefore needed that can combine two signals using an embedding operation to prevent the listener of the cover audio file detecting the change, while also taking into account the aforementioned limitations to meet the requirements of smart devices. The main consideration is that the secret data should be undetectable by the listener: the other signal, known as the cover signal, should be the only signal heard.

We therefore developed a low-cost method of randomly embedding scrambled text messages into speech signals before sending them over the Internet, based on the use of a hyperchaotic generator algorithm. There are two main phases to the proposed system. To deliver scrambled text during the first phase, we convert text characters into ASCII code before storing them in a vector. After slicing the vector into three parts, a backward-forward reading algorithm and odd-even indices (low-complexity operations) are used to scramble it. Two scrambling loops are considered: one for the small sub-vectors, and one for the vector as a whole.

The next phase involves the use of a hyperchaotic steganography approach that relies on low-complexity hyperchaotic maps. We use quadratic map (QM) and Hénon map generators to randomly locate a suitable location for embedding data into host speech. Prior to this, the speech signal is separated into 256 blocks with only 200 values per block, to conceal the hidden text. To minimize the effect of the embedding operation on the host speech signal, a speech steganography system based on the lightweight conditional least significant bit (LSB) method is applied to embed the confidential message into the LSB of the fractional portion of the speech signal amplitude value. By combining hyperchaotic maps with conditional LSBs, we create a steganography method that makes data recovery extremely challenging for an attacker.

The problem statement studied in this work is characterized as hiding the secured transmit user’s text in a sequence speech signal before sharing it on social media so that the received user can restore the secret text in a simple recovery method.

The fundamental goal of this work is to present a lightweight method that can hide information within .wav files, while maintaining the quality of the stego-speech samples, and which can conceal a significant number of text message characters in a secure manner. We combine scrambling and steganographic techniques to apply two levels of information protection. The use of a hyperchaotic approach for highly random insertion means that finding the random hiding locations inside the cover is difficult. In addition, the combination of lightweight hyperchaotic maps with steganography based on the QM, the Hénon map, and the LSB approach in this study is helpful in achieving low complexity and a secure hiding method. To the best of our knowledge, our scheme has a lower computing complexity than other related work, meaning that it could potentially be adapted for use with lightweight intelligent devices such as mobile phones and tablets. Therefore, the motivation of this study is to apply hyperchaotic techniques and conditional LSB in the audio steganography field to hide scrambled encrypted messages with a large capacity and a low-complexity algorithm while staying safe.

The paper’s contributions involve the following:

1. A new low-cost technique for scrambling data focuses on binary representation, making it difficult for a hacker to retrieve the data.
2. A speech signal steganography algorithm based on a low-complexity hyperchaotic map (QM and the Hénon map) and the LSB substitution technique.
3. Given the higher embedding capacity, stego speech quality is acceptable.
4. The performance of the proposed system is assessed using three datasets, and our technique is reviewed and compared with comparable work to identify its strengths and drawbacks.

This paper is divided into the following sections. Section 2 discusses related work in the area of steganography. The proposed method is introduced in Section 3. Section 4 presents some results and a performance evaluation. Finally, Section 5 concludes the paper.

## Section 2: Related work

Over the years, scientists have devised various methods of concealing sensitive information by creating hidden messages that are embedded indirectly in the time domain under an audio cover. They have also established various transformation strategies to alter the domain. In this section, we discuss the most recent approaches towards steganography in audio files. We then describe the security and durability of these methods of hiding secret data in an audio file to render it inaccessible to hackers [27].

The technique suggested in [3] involves scrambling a text message using a quantum chaos map, encoding the input text message with a chaotic map (called a Zaslavsky map), and then implementing the LBS method with indexing using a K-means algorithm to embed the encoded text into a voice signal. The size of the cover file remains constant when the encrypted message has been hidden in the voice file. This approach was used to combine various texts using text content with different lengths within the same speech samples. The results were encouraging: the text message was not lost, and there was no noise in the cover media. However, the K-means algorithm requires a considerable amount of time, as does the overall algorithm, and its presence may differ when using the same audio sample with a slight variation, exposing the hidden data to loss.

The work in [15] described a secure system for hiding an audio signal based on two stages: in the first, an encrypted message was secured by bit cycling, while in the second, the text message bits were randomly hidden in an audio file using an advanced LSB algorithm. As a result of limitations on the number and size of text messages, the proposed system was not compared to other previous systems in terms of its operational efficiency.

The study in [20] described an audio data-hiding technique that used a modulus function-based 8 to match the secret octal digits with the remaining values of the sample. In this technique, the cover sample was removed to calculate the difference between the undetected digits and to sample the remaining values. The remaining stego-sample then became the precise target secret octal digit. The audio was constructed with a function called “uint”, in which the binary base was not equal to the bit depth of the original audio cover. This meant that the quality of the stego-file did not meet the security requirements for data hiding.

The authors of [21] attempted to improve the capability of the steganography technique and to investigate the quality decline caused by the use of traditional LSB, and made specific modifications to the traditional LSB method. They found that the risk of quality deterioration decreased with the size of the cover file, whereas noise increased with the size of the concealed message. This technique resulted in no major improvement, as it was simply a direct extension based on increasing the number of bits used in the LSB algorithm to hide information.

One suggested method that relies on both cryptography and steganography techniques used the genetic algorithm [22]. A key was created randomly using a genetic algorithm during the encryption stage, and was then used in the one-time pad procedure. The encrypted message was then inserted into an audio file at the optimal embedding position using the SNR ratio of the evolutionary algorithm fitness function. The suggested scheme eliminated the disadvantages of key generation and distribution for the one-time pad by employing a genetic algorithm. Based on the results of experiments, the proposed method was reported to offer superior security and audio quality compared to numerous other steganography techniques. When used in real-time audio steganography applications, however, the time analysis may be excessive, as the computational complexity of a genetic algorithm increases the time required to implement the embedding algorithm process.

The authors of [23] inserted text messages into .wav files using the LSB technique. The 4-wrap length technique was used to determine the insertion position of the message inside the cover in order to randomly create insertion results so that the message’s contents could not easily be recognized. As a cover, a sample of audio data files with a duration of 10 s were used, with five text messages of varying lengths. The researchers found that the larger the text content, the higher the MSE and the weaker the peak signal-to-noise ratio (PSNR). However, it could also be observed that the MSE and PSNR values were not at the required level.

The study in [24] described a strategy for speech steganography based on the parity-segmented method and differential singular value decomposition (SVD). The discrete cosine transform (DCT) coefficients were separated into two segments based on parity order. Data embedding minimized the changes in singular values caused by partitioning the segments with roughly equal energy. As a result of modifying the difference between the most significant singular values, the adaptive embedding threshold was determined by the two most significant singular values. The proposed method achieved better imperceptibility, robustness, and security; however, the main limitation of this work was that the SVD transformations required a matrix with two main channels, meaning that the proposed method could not be used with single audio or speech input files.

The study in [28] described a mixing four Discrete Wavelet Transforms (DWT) and One-Time Pads (OTP) is robust for securing images versus hidden information. This method can guarantee double security for secret image exchanged over the World Wide Web using a combination of steganography and cryptography. No attacker can decipher the ciphertext encrypted by OTP, even if they have limitless computational power. In practice, OTP is impractical since it requires an encrypted transmission path.

The study in [29] presented a technique for growing secret messages utilizing division and modulus functions with the goal of expanding the message capacity encoded in the digital image based on LSB. Because communications are divided into two portions and delivered independently, the division and modulus function help increase message security. This study found that embedded messages can be doubled in capacity while maintaining imperceptibility, but the process may take longer.

In [30], a simple and successful method was described for hiding data in audio files using 256-bit AES encryption and the LSB algorithm. When the suggested technique was used, it was found that the stego-carrier had no discernible noise. The size of the output stego-carrier was also found to be virtually unchanged in file size, which is important, as a large file size may raise suspicions and lead to the loss of sensitive data. This methodology was not evaluated for efficiency using any metric other than the time taken to hide the text messages of different file types, such as.txt, .pdf, and .ppt. The time taken was relatively high.

The authors of [31] suggested technology for audio steganography that could lead to superior steganographic cover audio for automatic message embedding. The proposed framework’s training architecture consisted of three components: a generator, a discriminator, and a taught steganalyser, based on deep learning. As part of this process, the LSB matching (LSBM) approach was used to embed the secret message in the steganographic cover audio. The result was then submitted to the trained steganalyser for misclassification as cover audio. These three parties were able to develop steganographic cover audio generators for the embedding of messages after adversarial training. The results of their experiments indicated that audio steganography could provide steganographic cover audio with good perception quality for encoding messages, but that it took longer to train the network used in the deep learning-based steganalyzer.

The system in [32] combined the two techniques of steganography and encryption. AES was used to generate ciphertext after encrypting the communication. The ciphertext was subsequently transformed into audio signals using steganography and the LSB algorithm. Since the audio recording was in ASCII format, the contents of the ciphertext were converted to a bitstream. The encoded record was separated from the audio file at the receiver. This approach was a traditional one, and no metrics for system efficiency, concealed storage capacity, or cover audio file amount were considered.

In [33], a message was hidden in an audio file before being delivered, and the recipient retrieved it. In keeping with the psychophysiological model of sound perception, the steganography system used compression to enhance the integrity of audio and text information. The text information was adaptively normalized using a discrete wavelet transform, followed by recursive embedding in the low-frequency components of the audio signal, which were then scalar-produced using Daubechies wavelet filters. The amount of information that could be incorporated into an audio signal with equivalent qualifications was comparatively small, due to the increasing inaccuracy of the wavelet decomposition. However, the outcome of this strategy could be highly successful if the audio container was not used to hide a significant amount of data.

The study in [34] proposed a strategy based on the discovery that the power of energy compaction with DCT increases when applied to homogenous data, thus creating a large embedding capacity. The proposed hiding strategy therefore segmented the carrier voice signal into correlated segments to conceal the amplitude of inconsequential DCT coefficients. In this approach, a novel quantization function suitable for normalized speech samples was used to find the unimportant DCT coefficients. Several tests were applied to evaluate the influence of different cover speech signals in terms of gender, age, language, and accent, but a clear comparison of the time cost of hiding and restoring text messages could not be carried out.

In [35], scrambling and steganography were combined to provide a high level of privacy for the transmission of hidden information. By using super-Gaussian signals as a basis, the scrambling block adapted speech signals to super-Gaussian signals. The security of a super-Gaussian signal depends on its seed value; once scrambled, the speech signal is hidden in a non-sensitive speech signal, and the responsive hiding process is defined by the number of bits to be held (BH). The recovered signal appeared to be the same as the original secret message, meaning that in this method, the host signal had a negligible impact on the quality of the recovered signal. However, the problem of exposing the speech signal to imperceptible noise remains a significant factor in terms of changing its characteristics, and may affect the correct recovery of hidden information.

Based on a multiple-layer discrete wavelet transform, the researchers of [36] designed an approach for steganographically concealing text information in audio signals. The experimental outcomes confirm that it is proposed to use recursive embedding in the approximating wavelet coefficients followed by their scalar product with wavelet filters at each level in the discrete wavelet decomposition to increase the average power of hidden data. Because the error increases with each subsequent level of discrete wavelet decomposition, the quantity of information that could include in an audio signal with equivalent quality will be much less than in present systems. As an outcome, it must stress that this strategy will be highly successful if a small quantity of data is not hiding in the audio signal.

[31] suggested utilizing the generative adversarial network (GAN) to produce better steganographic protection audio. The chosen method’s training framework consists of three major modules: a generator, a discriminator, and an off-the-shelf deep learning. When the adversarial training among these three parties is done, one can receive a well-trained generator capable of producing steganographic cover audio for future message embedding. Using the well-trained generator, one may incorporate secret messages like traditional steganography. The experiment results indicate the proposed steganography can provide steganographic cover audio with excellent perceptual quality while preserving relatively good undetectability performance, even at high embedding rates. The disadvantage is that the production and reconstruction modules require prior knowledge of the hidden audio. If a new secret audio is provided, the suggested technique must be shown again on the original base.

In [37], authors suggested a hybrid system for image hiding based on LSB replacement and enhanced modified signed digit (EMSD) procedures. LSB replacement is used as a way to improve EMSD. The approach controls the capacity and physical concealment by varying the number of neighboring pixels and LSBs.

Scientists at [38] offer a two-step steganography-based data concealing system that improves the quality, capacity, security, and efficiency of embedded stego-image safety. The hidden information is encrypted and relocated in order to ensure privacy. To conceal encrypted data in the cover image, researchers suggested using LSB+3 types I and II. With an excellent level of reliability, this approach gives additional security to image steganography.

Over the previous two decades, the utilization of multimedia devices has expanded to include a wide range of applications, with a greater emphasis on digital audio data security and secure communications. This study will explain how to employ a highly redundant audio signal obtained from speech microphones as a frame to hide scrambled text information within, thus solving the traditional challenge of speech steganography. Voice messengers, for example, could use the developed approach to conceal scrambled text messages with phony audio messages so the attacker will find it hard to locate the base of the users’ private correspondence.

We analyzed the related works described above and considered their limitations to assess the contribution of our own work to the field in terms of generating stego-cover audio files (.wav). Our method is lightweight, with excellent perceptual quality, and the hidden message (.txt) is placed into a complicated situation using a low-cost scrambling technique and random algorithms implemented in binary representation format. The proposed scrambling algorithm is simple, has a low cost, and relies on binary representation scrambling, thus guaranteeing that it is difficult for a hacker to recover the data. The binary representation inserted a difficulty restoration method by dividing the text message into random mixed parts. This process is repeated after the text message has been collected, before it is converted to ASCII. In order to reduce the distortion caused by data masking, the speech signal is divided into equally sized blocks, to ensure that the hidden text data are distributed evenly across the speech samples. A hyperchaotic map and a conditional LSB algorithm are developed for the insertion of text message bits. The hyperchaotic map is based on the low-complexity QM and the Hénon map, which are used to randomly identify the location for the insertion of the message inside the cover. Finding these randomly selected concealed locations to recreate the correct sequence therefore becomes difficult, making the recovery of concealed data harder and a greater challenge for an attacker. Due to the lightweight nature of the LSB technique and hyperchaotic maps, the implementation time is low, meaning that the requirements of smart devices can be met. The performance of the proposed system is determined using three datasets, and our method is evaluated and compared with related work to identify its strengths and weaknesses. The SNR, MSE, structural similarity index measure (SSIM), PSNR, mean opinion score (MOS), BER, and perceptual evaluation of speech quality (PESQ) methods are applied as described in Section 4 (Tables 2–6 and 8) to evaluate the steganography and audio standards before and after addition of the message.

## Section 3: Methodology

In this study, speech steganography was carried out in two phases: text messages were first scrambled, and then randomly hidden within speech signals. Each stage of the process was undertaken using different methodologies. For the scrambling phase, we developed a method of dividing the vector values of the entered text in binary form into three vectors, and then mixing them in a manner that made them difficult to retrieve. For the hiding phase, we used hyperchaotic maps to randomly select locations for conditional LSB steganography. The hidden message was retrieved using the same operation in reverse.

### Chaotic map

Many scientists worked on chaos theory in the 1970s. Matthews was the first to suggest the use of a chaos-based encryption algorithm, and following this idea, numerous chaos-based image encryption schemes were proposed and tested [39]. Chaotic algorithms have been implemented in many steganography applications. Chaos dynamics offers a clear understanding of impromptu behavior in some nonlinear dynamical systems, and has been used in security methods to diffuse information [19]. A chaotic system has many attractive features, such as sensitivity to the initial conditions, control parameters, ergodicity, the mixing property, and quasi-randomness [40].

A hyperchaotic system needs to have no fewer than two positives exponent Lyapunov numbers and one zero value to ensure the variety and complexity of its behavior. A hyperchaotic system must display extensive and complicated bifurcations as the system parameters vary to demonstrate extremely unpredictable behavior. Because a hyperchaotic system lacks a stable balancing point, it might display random and unexpected behavior [41].

The hiding procedure in the proposed system is based on a technique for generating hyperchaotic keys using two types of chaotic maps (here, the quadratic and Hénon maps), which provide the locations of where the scrambled text data are to be randomly hidden before covering them with the LSB. These two chaotic maps were used in our work in view of their low computational cost [42–44], and are described in more detail below.

#### Quadratic map

Quadratic functions were created by modifying functions. The first study of a QM was carried out in the early 1970s. The QM is a chaotic one-dimensional map that converts real numbers from the range [−2, 2] to the same range. Depending on a variable ∂, these characteristics can change dramatically [45].

The quadratic map is expressed as shown in Eq 1 below [46]:

$$\begin{array}{c} Q\left(n\right)=\partial +{\left(u-Q\left(n-u\right)\right)}^{2} \end{array}$$ (1)

where n is the number of iterations and u is the additive element of chaos, when ∂ ∈ [0, 2], *Q* ∈ [0, 1] in which the chaotic is realized when ∂ ∈ [1.5, 2] [46].

These constants must be determined and their reliability evaluated in order to ensure that good information is available about what is happening within the QM. Although the initial frequency varies only a little, the QM has auto- and cross-correlation properties that are similar to those of arbitrary white noise. The quadratic chaos generator has good autocorrelation properties, making it suitable for use in security applications [47].

*Remark 1*. The use of one-dimensional quadratic chaotic maps to generate chaos keys, which are then used to determine random scrambling locations for hiding text message bits in speech signal bytes, has several disadvantages, including simple chaotic actions and limited key space. A more complicated chaotic quality indicates that more than one chaotic map was used or was mixed in hyperchaotic keys. Consequently, in our study, we rebuilt a 2D Hénon map using essential parameter values from a 1D quadratic chaotic map.

#### Hénon map

The Hénon map is one of the most widely studied aspects of chaotic discrete-time system dynamics. It was devised in 1978 [48], and is expressed as shown in the following Eq 4:

$$\begin{array}{c} H_{m+1}=1-\omega H_{m}^{2}+L_{m} \end{array}$$ (2)

$$\begin{array}{c} L_{m+1}=\phi H_{m} \end{array}$$ (3)

where *ω*, *ϕ* are two bifurcation parameters, *ω* > 0 and *ϕ* > 0, and $H_{m}^{2}$ is the seed map [4].

This expression exhibits chaotic behaviour for *ω* ∈ (0.54, 2) and *ϕ* ∈ (0, 1) [49]. When *ω* and *ϕ* are 1.4 and 0.3, respectively, the system is chaotic and takes the form of the classical Hénon map; for other values, the map may be chaotic, unreliable, or may converge to a periodic orbit [48].

In our strategy, the two variables used to create a Hénon chaotic map, *ω* = 1.4 and *ϕ* = 0.3, are adapted to generate an index key to detect the areas in which we have hidden the bits of the message in the audio signal.

*Remark 2*. The Lyapunov exponent is extremely sensitive to minor variations in the initial parameters, such as the initial conditions and control parameters of the 2D Hénon map. In this variation of the Hénon map with a 2D structure, the Lyapunov coefficient and scrambling efficiency of the cover speech signal can be increased to enhance the effectiveness and number of parameters during generation of the hyperchaotic key.

### Least significant bit

The LSB is a simple steganographic procedure employed to conceal scrambled text in different multimedia data. The LSB embedding algorithm replaces the LSBs of the cover multimedia with the bit stream of the scrambled text to be hidden [2]. Due to the noise present in the multimedia, this process only changes the least significant bits of the original multimedia, and never the most significant bits (MSBs) [5]. This approach uses a maximum data transfer rate of 1 kbps per 1 kHz, which means that one LSB covers one bit of hidden data. Over a few executions of the LSB code, the two LSBs are substituted with two data bits to increase the amount of information concealed in the stego-audio file [9]. This scheme is an effective method of increasing data coverage, and is relatively simple to implement, but is vulnerable to attacks [20]. We therefore introduced conditional instructions and randomization when locating the information steganography, to enhance the attack resistance of the LSB algorithm.

*Remark 3*. The internal conditions depend on the other bits in the cover speech signal to hide inserted text message bits rather than only the LSBs in the LSB algorithm. This ensures that hackers are prevented from identifying the hidden values in the audio samples. A condition based on the MSBs was used to establish criteria for determining the location of the hidden data in one of the last two LSBs. As a result, the conditions added to the LSB method to determine which bits are used from the LSBs to hide the scrambled text message was a difficult modification to avoid detecting the value and location of the hidden data. A low-cost hyperchaotic map is used to identify the location of the message hidden inside the audio cover.

### Proposed method

The proposed algorithm is divided into two steps. The first is the text scrambling algorithm, which involves converting the ASCII characters of a text message into a binary representation and saving them in a vector; this vector is then divided into three sub-vectors and the values in each sub-vector are scrambled using low-cost techniques (the odd-even index and forward-backward reading techniques). The scrambling circuit can be expanded from tiny sub-vectors to larger ones by performing this method on the original vector to further enhance security.

**Fig 1:**
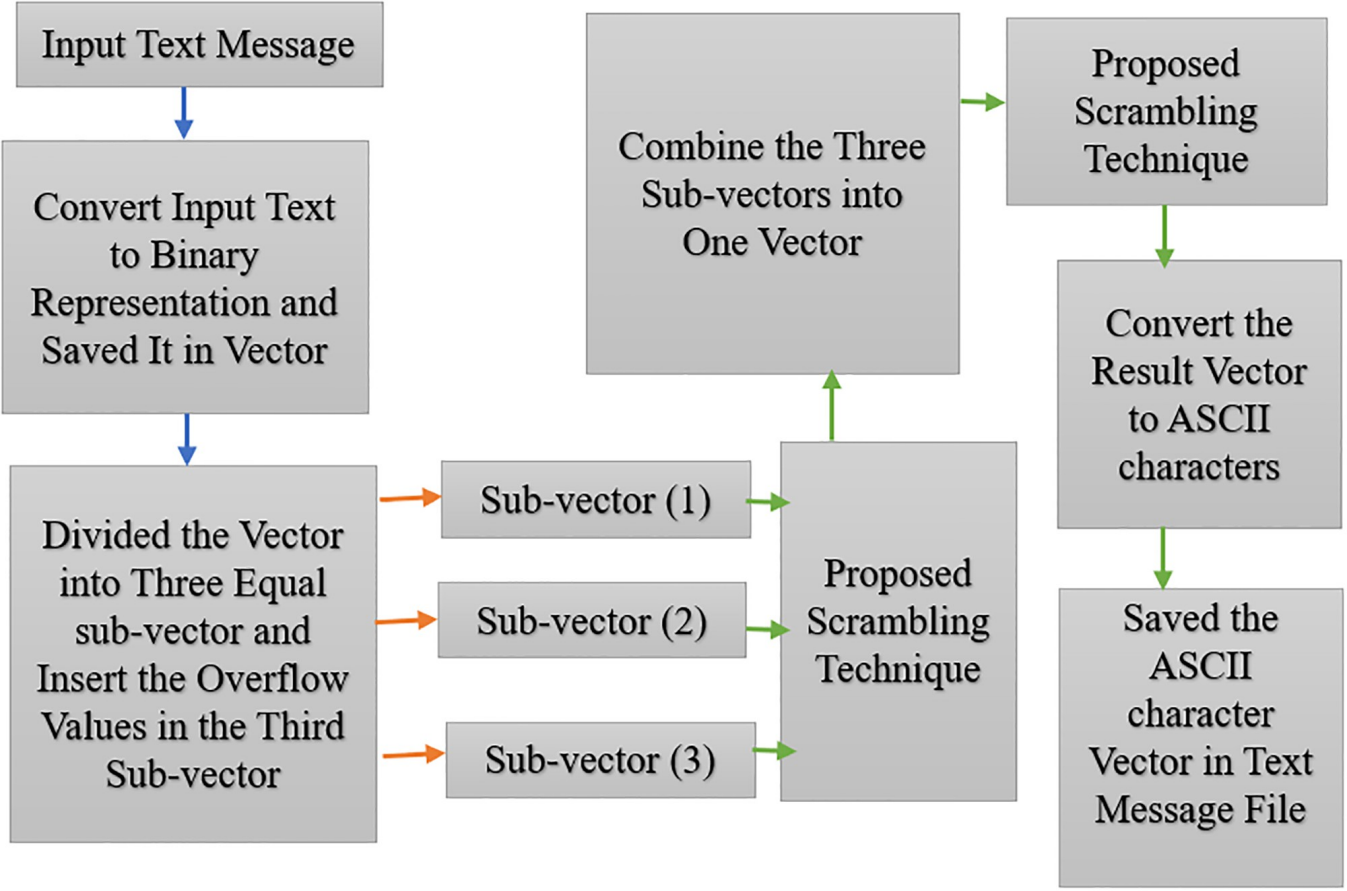
Diagram showing the proposed scrambling process.

**Fig 2:**
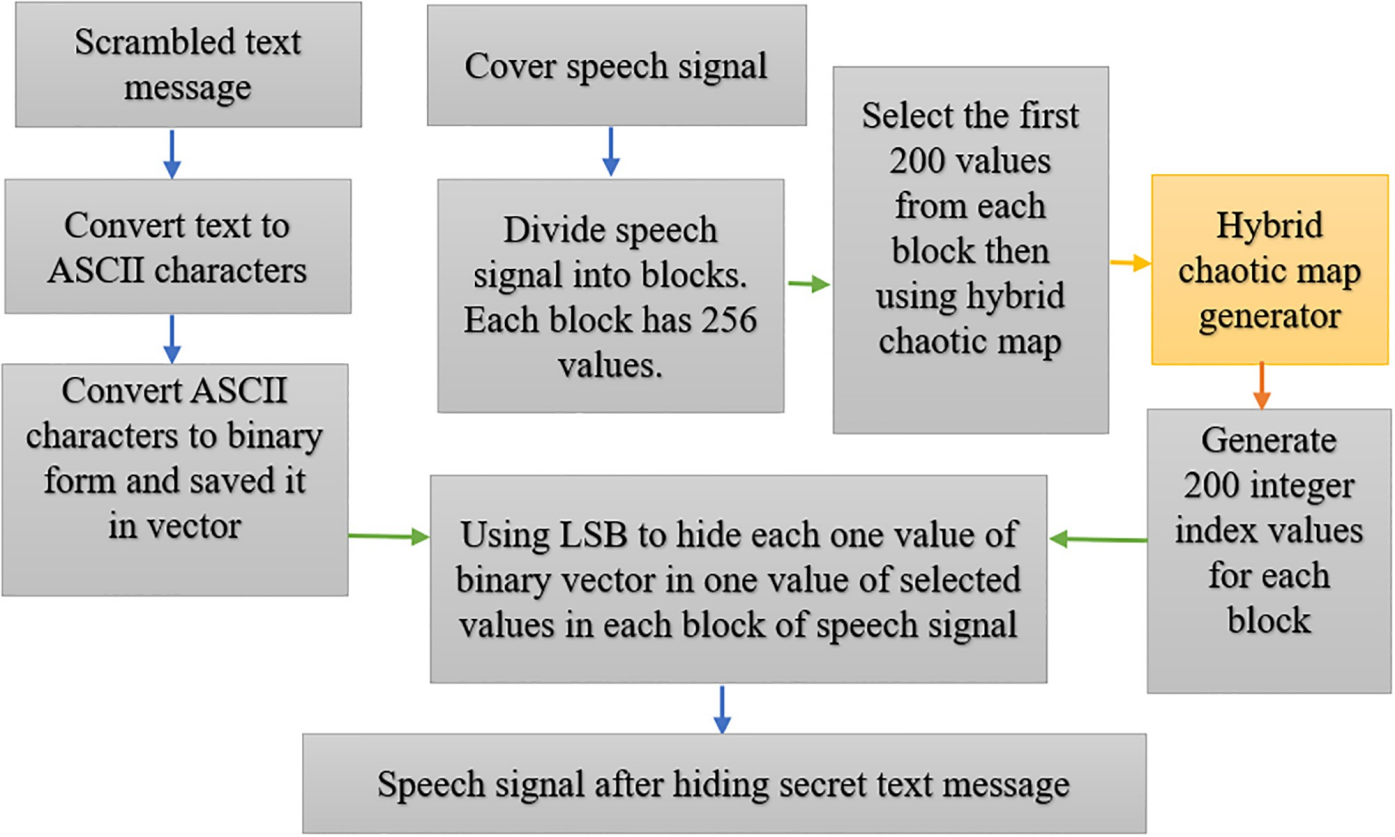
Diagram showing the embedding process of the text message.

The second stage is the hiding algorithm, which conceals the scrambled text bits in the speech samples. The speech samples are divided into blocks containing 256 values each, although only 200 are used. We then use the low-complexity hyperchaotic map, based on the QM and the Hénon map generator, to generate an index key to detect the areas where the text bits are hidden in the speech data. The locations in the speech file where the bits are hidden are chosen using the LSB as a lightweight method. As a result, the suggested method is a more secure and complex way of hiding data, and can prevent hackers from detecting the concealed text, as shown in Tables 2, 4, and 6 in Section 4. Figs 1 and 2 illustrate the proposed method.

#### Proposed scrambling method

This algorithm starts by reading text messages saved in .txt files, and then converts each text message from its ASCII form to binary form and saves its values in a vector containing digits one and zero. The vector is then segmented into three sub-vectors of equal length, with the excess of the original vector length stored in the last section. The proposed lightweight scrambling algorithm is applied at the binary representation level to ensure that the values are mixed in a complex manner that makes it hard for attackers to retrieve the data. Our low-cost technique for scrambling begins with the odd-even index strategy, which involves swapping each odd index location with an even index location. This is followed by the forward-backward reading procedure, which saves the resulting vector of values in reverse by reading the background of the three-piece vectors and saving them based on forward indexing. Finally, the same action is applied to the resulting vector after merging the three sub-vectors once again, as seen below in Fig 1 and illustrated in Algorithm 1. The scrambling operations used here are simple and effective, and the computational costs are kept as low as possible.

**Algorithm 1**:

**Begin**:

**Input**: Input text message.

**Output**: Scrambled text message.

**Steps**:

**Step 1**. Convert the input text file to a binary format and save it as a vector T1.

**Step 2**. Divide the binary form vector into three pieces, keeping in mind that the last vector stores more values if necessary.

**Step 3**. Complete the following steps for each of the three pieces:

 a For all values in the vector, replace the values in the even and odd locations.

 b Create a new vector by reading the values in the previous vector twice, once from the far right and once from the far left.

**Step 4**. Combine the three pieces into a new vector and, this time, repeat the whole of step 3 on the new vector.

**Step 5**. Save the new vector and convert it from binary representation to ASCII code, which will subsequently represent the text message following the scrambling process.

**End**

#### Hyperchaotic generator

This section explains how the hyperchaotic map is created. The design is based on two standard low-cost chaotic maps: the QM and the Hénon map. This approach has a particular sensitivity to the initial conditions and parameters. The process begins by using Eq 1 to produce the key N using the QM, and the results are passed as input to Eq 2 once instead of the L value, and once by using Eq 3 to generate the L value. The values of the Hénon map distribution are integer numbers in the range [0, 200], with no repeats, as index detection where we hide scrambled text bits.

#### Hiding process

The hiding method relies on a hyperchaotic map and LSB algorithm to randomly hide scrambled text messages in a speech signal. The algorithm begins by reading the scrambled text message and converting it to binary representation in order to save it as a vector. The speech signal is divided into blocks, each with a fixed size (256 values), in order to distribute the hidden data bits evenly over the blocks of the speech samples. We note that only 200 locations are used from each block in order to ensure that data recovery is difficult. Using the hyperchaotic map generator, an indexing value ranging between one and 200 is generated. After converting the fractional values of the specified location into binary form, the specific bit is embedded based on a conditional set applied to the LSB algorithm. We select only eight bits from positions 17–24 for each location, as it was discovered from experiments that these locations had a minimal influence in terms of distorting the speech signal. After the embedded bit has been hidden, the value is converted back to fractional form and a new speech signal is restored, giving the waveform after embedding, as shown in Fig 2 and described in Algorithm 2.

**Algorithm 2**:

**Begin**:

**Input**: Scrambled text file, cover speech signal. Stego-speech signal.

**Steps**:

**Step 1**. Read the scrambled text file, transform it from ASCII format to binary form, then save it in vector format.

**Step 2**. Read the speech signal values, and split them into blocks of 256 samples.

**Step 3**. Using the hyperchaotic map generator, create a series of unrepeated random integer values with a length of 200 values, which is the number of samples chosen from each block to hide text message bits in.

**Step 4**. For each speech signal block, carry out the following:

 a Determine the location using the hyperchaotic map generator.

 b Break the bits between positions 17 and 24 of the 64 bits to each number in binary form, and then apply the conditional LSB algorithm to hide the bits of the secret text in the last bit (b1). We assume the bits have the following format: b8 b7 b6 b5 b4 b3 b2 b1. Bits b8, b7, b6, and b5 are called the most significant bits (MSBs), and the remainder are the least significant bits (LSBs).

 c Carry out an XOR operation between b8 and b7 and save the result in a variable called R1.

 d Carry out an XOR operation between b6 and b5 and save the result in a variable called R2.

 e If R1 is equal to R2, obtain the value of b1 to the detected scrambled text message bits. Otherwise, replace the estimated scrambled text message bits in b2.

 f After concealing the text data, reconnect the 8 bits that were clipped out from the 64 bits.

 g Change the 64 bits of binary data into fractional numbers to get the elements of the incoming speech samples.

**Step 5**. After the hiding process, merge the speech sample blocks into a single vector that represents the output speech sample vector.

**Step 6**. Insert the length of the secret message into the last block minus one of the speech samples to tell the receiver how long the scrambled message is.

**End**

#### Extraction process

The secret text message can be retrieved using a similar algorithm to that used for embedding. The steps are described in Algorithm 3 below.

#### Reverse scrambling process

This algorithm retrieves the text message by implementing the techniques applied in the scrambling algorithm in reverse. First, the ASCII code representing the scrambled text message is converted to binary representation and saved in a vector, which is then divided into three pieces of equal length. If there are extra values, these are added to the last piece. The algorithm then reads the elements of each vector in reverse, from back to front. Next, the values in the odd and even locations are swapped. Finally, the contents of the three vectors are merged into a single vector that represents the original retrieved text message. The reverse scrambling algorithm must first be applied to the entire scrambling message vector using two techniques, the odd-even index and forward-backward techniques, before it is segmented into three vectors to allow the text message to be recovered correctly. This reverse operation has a very low computational cost, meaning that our scheme is inexpensive in terms of hiding and extracting data.

**Algorithm 3**:

**Begin**:

**Input**: Stego-speech signal.

**Output**: Scrambled text file, Cover speech signal.

**Steps**:

**Step 1**. Segment the steganography speech samples using the same dividing length as the embedder (256 samples).

**Step 2**. Create a sequence of unrepeated random integer values with a length of 200 using the hyperchaotic map generator.

**Step 3**. For each block of speech samples, do the following:

 a Determine the location using the hyperchaotic map generator.

 b Split the bits between positions 17 and 24 of the 64 bits to each binary number.

 c Carry out an XOR operation between b8 and b7 and save the result in a variable called W1.

 d Carry out an XOR operation between b6 and b5 and save the result in a variable called W2.

 e If W1 is equal to W2, restore the value of b1 and save it in a vector containing all the detected scrambled text message bits. Otherwise, restore the estimated scrambled text message bits in b2.

**Step 4**. Convert the values in the vector from binary representation to ASCII code. Then save the resulting ASCII code vectors in a .txt file as the scrambled text file.

**End**

#### Discussion

The algorithms presented above were designed to hide the bits of a scrambled text message with limited alteration to a cover speech signal, with a low computational cost to make them suitable for devices with limited resources. The text message is scrambled using lightweight strategies including an odd-even index swap and a forward-backward process, which are applied to the binary form to make the recovery of the message more challenging. This process hides the scrambled message bits in random positions, using a low-complexity hyperchaotic map and a lightweight conditional LSB method, based on the MSB values. Two processes must be known in order to extract the secret message: the first is the process used to generate hyperchaotic sequences based on the QM and the Hénon map, while the second is the conditional LSB method, which is needed to correctly restore all the message bits. Consequently, this is a complex method of concealment that makes retrieving the information extremely difficult.

## Section 4: Experimental results

An experiment was carried out on three datasets. First, we considered 25 English speech samples in .wav format. As a cover, each file was a single-channel 16-bit pulse code modulation (PCM) with a sampling rate of 22.050 kHz and a duration of 4–10 s [3, 50]. Secondly, we considered wave audio files with a resolution of 16 bits, a sampling rate of 48 kHz, and a stereophonic sound system with two audio signal channels [51]. Finally, we used the TIMIT database, containing 6,300 utterances by 630 adults, speaking 10 sentences each in eight major dialects of American English [52]. The length of each speech file was approximately 1.4–5.04 s. In addition, text files (.txt) of various lengths were chosen randomly from the dataset described in [53]. Our method was implemented and tested on a computer with the following system and software specifications: Pentium Intel (R) Core i7, CPU@2.60 GHz, 6.00 GB RAM, 64-bit Windows 11 system software, and the MATLAB R2020b program. We chose a .wav file as the cover audio for embedding the data because it contains significant data redundancy, thus allowing for a greater amount of data to be hidden, as we employed an LSB technique that relies on redundancy to hide the data.

### Inaudibility

Inaudibility means that the secret message is not able to be heard. The message embedded in the host signal is inaudible. In this section, various metrics were used to evaluate the quality of the stego-speech. Six metrics were considered: the MSE, PSNR, SNR, SSIM, PESQ and MOS.

#### Mean square error

The mean square of the difference between the original audio and stego-audio signals is called the MSE, and can be used to evaluate the audio’s accuracy. When this value reaches zero, the quality of the original and stego-signals is equal, resulting in improved performance of the algorithm. Eq 4 was used to calculate the MSE as follows [1, 23, 54]:

$$\begin{array}{c} MSE=10\;{\text{log}}_{10}\sum_{i=1}^{m}\sum_{j=1}^{n}\frac{{\left(p\left(i\right)-d\left(i\right)\right)}^{2}}{M\times N} \end{array}$$ (4)

where:

*n* and *m* are the numbers of rows and columns in the cover speech signal; *p* is the sample with index number in the original speech signal; *d* is the sample with index number in the stego-speech signal.

#### Peak signal-to-noise ratio

The PSNR is obtained by averaging the audio quality before and after the text is embedded, and is measured in decibels (dB) [23]. The higher the value of the PSNR, the better the invisibility of the text file, and thus the higher its quality [54]. The quality rating limit is 30dB, and audio with PSNR higher than 40dB is considered good. The PSNR formula is given in Eq 5 [1, 27, 55]:

$$\begin{array}{c} PSNR=10\;{\text{log}}_{10}\left[\frac{\text{max}{\left(p\left(i\right),d\left(i\right)\right)}^{2}}{abs{\left(p\left(i\right)-d\left(i\right)\right)}^{2}}\right] \end{array}$$ (5)

#### Signal-to-noise ratio

SNR is a measure of signal strength in comparison to noise [31]. As a result, it measures the quality of the output signal after embedding the text file, and is measured in decibels (dB). A higher SNR indicates that the embedded data is more invisible [54]. SNR is calculated using Eq 6 [22]:

$$\begin{array}{c} SNR=10\;{\text{log}}_{10}\left(\frac{\sum_{i=1}^{n}p{\left(i\right)}^{2}}{\sum_{i=1}^{n}{\left(p\left(i\right)-d\left(i\right)\right)}^{2}}\right) \end{array}$$ (6)

#### Structural similarity index meter

The SSIM is used to represent the confidence of the interrelations in the speech. A value of the SSIM that is closer to +1 indicates a better result. The SSIM is calculated as shown in Eq 7 [3]:

$$\begin{array}{c} SSIM\left(P,D\right)=\frac{\left(2\sigma_{p}\sigma_{d}+\tau_{1}\right)\left(2\alpha_{pd}+\tau_{2}\right)}{\left(\sigma_{p}^{2}+\sigma_{d}^{2}+\tau_{1}\right)\left(\alpha_{p}^{2}+\alpha_{d}^{2}+\tau_{2}\right)}\times 100 \end{array}$$ (7)

where: *σ*_*p*_ and *σ*_*d*_ are the mean values of *p* and *d*, respectively. *α*_*p*_ and *α*_*d*_ are the standard deviation values of *p* and *d*, respectively. *τ*_1_ and *τ*_2_ are two constants used to avoid a null denominator.

**Fig 3:**
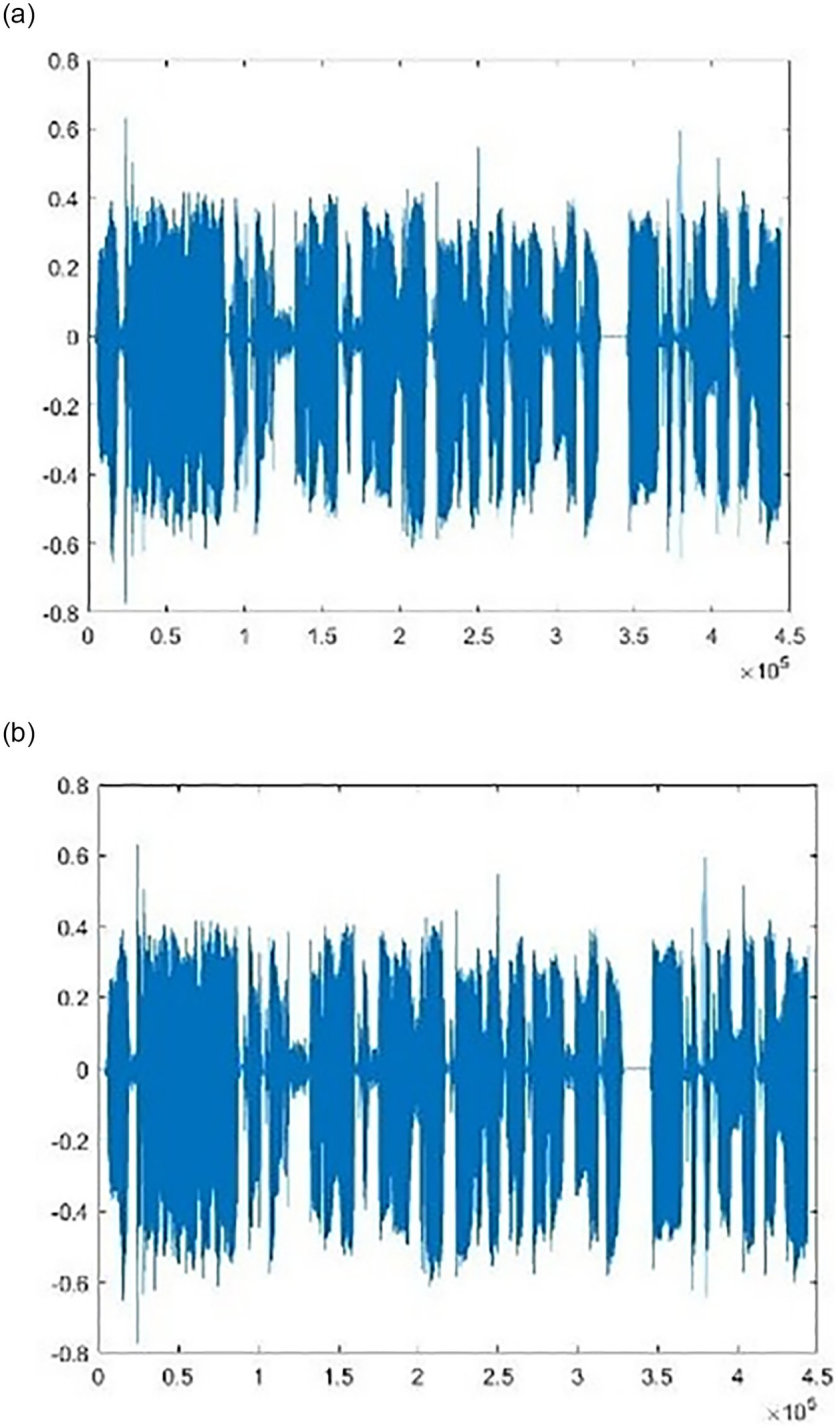
Speech signal waveforms (a) before and (b) after hiding the encrypted text message.

The period waveforms for a speech signal named “speech1” are shown in Fig 3, before and after a scrambled text message of 500 characters was embedded. It is clear that using the suggested approach causes no change in the signal.

**Fig 4:**
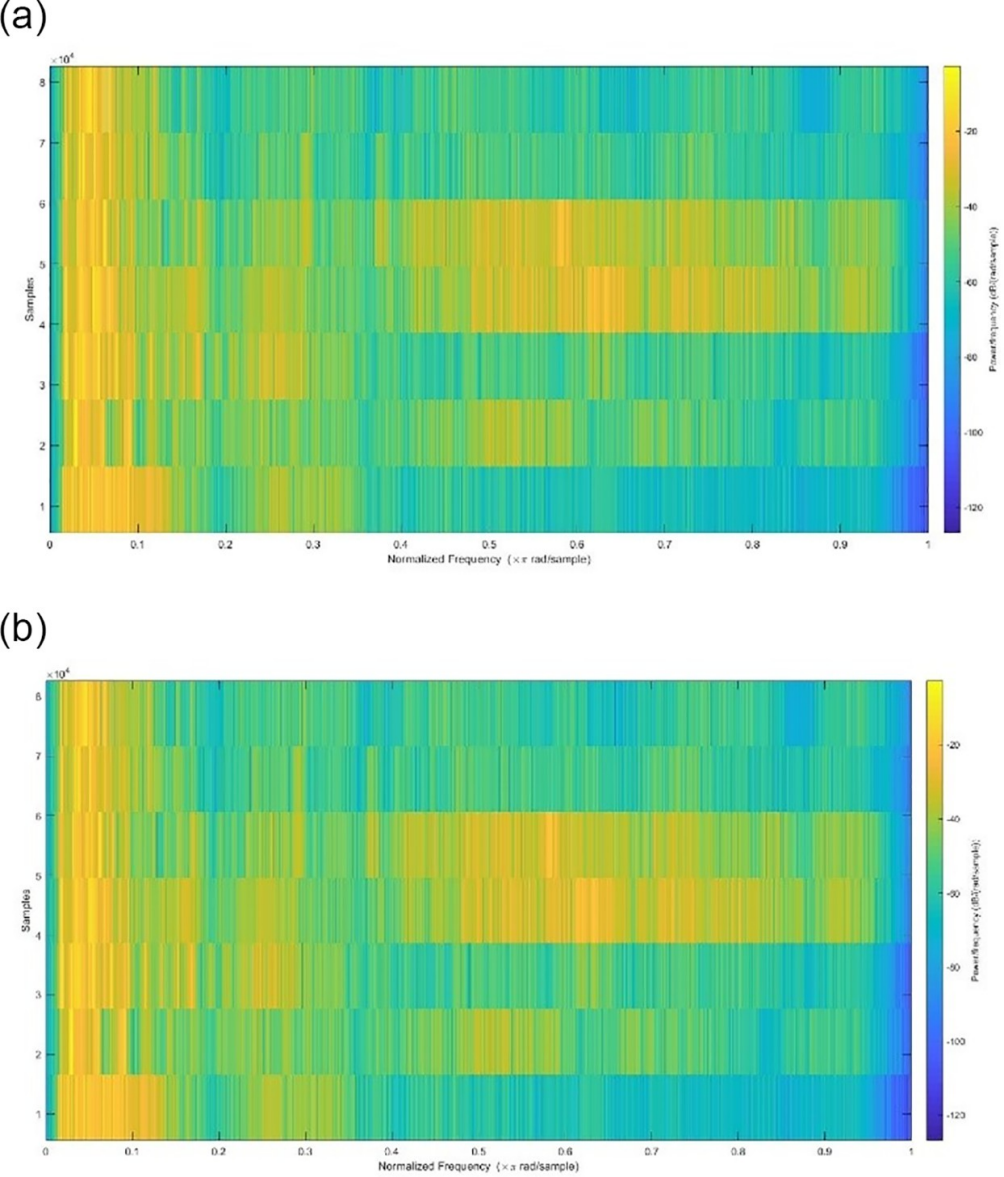
Spectograms of (a) the original speech signal, (b) the stego-speech signal.

Spectrograms of the original and stego-speech signals are shown in Fig 4, and it can be seen that there are no clear differences.

Tables 1–3 summarize the results for the performance of the proposed algorithm in terms of hiding text in a speech signal. Table 1 compares the proposed algorithms with other current approaches described in [3, 24], and [25] using files called “Speech1.wav”, “Speech2.wav”, and “Speech3.wav”. The various performance outcomes are determined using the different lengths of the message. According to the data, the lower the MSE value and the higher the SNR and PSNR, the better the stego-speech signal is generated. The SNR, MSE, and PSNR values achieved after hiding data with various message lengths in speech sample files are displayed in Table 1.

**Table 1:** SNR, MSE and PSNR values for different speech signal and text files after the hiding process.

Speech file (.wav)	Capacity (bytes)	Embedded (bytes)	payload capacity (BPB)	[3]			[24]			[25]			Proposed algorithm		
				SNR	PSNR	MSE	SNR	PSNR	MSE	SNR	PSNR	MSE	SNR	PSNR	MSE
Speech1	443926	323	0.07%	254.463	142.216	2.67E-09	258.234	171.254	2.17E-11	232.126	169.541	1.19E-10	**255.554**	**170.348**	**5.16E-13**
		836	0.18%	196.409	113.188	2.13E-09	191.91	155.451	3.03E-10	187.176	151.145	2.04E-09	**190.669**	**153.405**	**4.04E-12**
		4931	1.11%	176.72	103.344	2.06E-09	189.127	101.154	2.36E-09	171.874	98.1	1.54E-08	**187.172**	**100.157**	**1.35E-11**
		28959	6.50%	162.129	96.049	1.10E-09	163.365	93.212	1.05E-09	159.296	89.276	3.41E-07	**161.493**	**91.317**	**1.64E-10**
Speech2	571258	323	0.05%	256.036	143.662	2.46E-09	248.437	172.657	3.88E-11	244.397	169.743	1.86E-10	**244.263**	**171.963**	**4.72E-13**
		836	0.14%	197.529	113.909	2.32E-09	201.98	110.112	2.06E-10	194.348	107.12	3.04E-09	**198.005**	**108.334**	**3.96E-12**
		4931	0.86%	178.794	104.541	2.01E-09	165.101	100.786	1.12E-09	161.276	96.709	1.17E-08	**162.083**	**99.072**	**1.03E-11**
		28959	5.06%	163.606	96.947	1.15E-09	159.267	91.065	1.01E-09	153.9	90.581	1.04E-07	**158.34**	**91.001**	**1.54E-10**
Speech3	900412	323	0.03%	261.447	145.71	2.42E-09	266.876	179.276	4.57E-11	261.023	166.76	3.53E-10	**263.298**	**177.939**	**4.58E-13**
		836	0.09%	202.943	116.458	2.04E-09	209.112	111.126	2.10E-10	198.182	107.634	1.02E-09	**208.31**	**110.04**	**3.75E-12**
		4931	0.54%	182.794	106.384	2.07E-09	172.349	99.908	2.10E-09	169.376	93.9	2.11E-08	**171.518**	**98.949**	**1.00E-11**
		28959	3.21%	167.798	98.886	1.16E-09	159.212	91.098	1.31E-09	153.265	88.812	2.32E-07	**158.374**	**90.977**	**1.40E-10**

**Table 2:** PSNR and MSE performance for text messages of different lengths.

Audio file length (ms)	Text length	[3]		[22]		[23]		[24]		[25]		[26]		Proposed algorithm	
		PSNR	MSE	PSNR	MSE	PSNR	MSE	PSNR	MSE	PSNR	MSE	PSNR	MSE	PSNR	MSE
10	15	191.11	1.71E-13	180.28	1.01E-12	181.75	8.44E-13	199.31	2.10E-13	184.12	1.19E-13	185.36	1.92E-13	**197.1**	**2.72E-14**
	30	158.63	3.03E-11	147.02	3.38E-09	149.74	1.69E-10	159.64	5.09E-11	151.65	3.00E-11	153.71	3.28E-11	**149.88**	**1.80E-12**
	50	143.84	9.12E-09	131.89	9.23E-09	134.57	2.78E-10	144.71	7.03E-11	135.88	1.17E-10	139.79	9.15E-10	**141.08**	**1.37E-10**
	75	140.21	2.11E-09	129.02	2.21E-07	129.86	4.12E-08	141.02	4.29E-10	130.2	2.01E-09	134.23	2.19E-09	**129.33**	**8.22E-09**
7	15	180.68	1.33E-13	170.51	1.43E-11	173.61	3.12E-12	183.76	2.00E-13	177.69	1.32E-13	179.56	1.65E-13	**172.67**	**6.65E-13**
	30	147.62	2.69E-10	136.84	3.00E-09	140.92	1.02E-10	149.81	1.54E-11	143.63	2.63E-11	145.81	2.11E-11	**141.08**	**9.57E-10**
	50	134.9	5.04E-08	123.59	5.19E-07	125.33	2.65E-08	136.53	3.30E-10	128.95	1.13E-10	130.28	5.07E-10	**129.1**	**1.55E-09**
	75	129.17	1.89E-07	117.5	1.57E-06	119.01	1.77E-07	130.51	2.91E-09	121.11	1.85E-09	124.29	1.10E-09	**122.51**	**6.89E-08**
4	15	158.33	1.35E-10	144.93	1.47E-09	149.73	1.26E-10	159.59	1.59E-11	156.36	1.30E-10	157.11	1.32E-11	**157.8**	**1.17E-13**
	30	145.2	2.79E-09	131.48	2.48E-08	134.24	4.10E-09	148.36	2.40E-10	138.23	2.78E-10	141.28	2.80E-10	**145.25**	**2.10E-10**
	50	137.97	1.47E-09	124.24	1.58E-07	128.58	1.80E-08	139.01	1.57E-10	129.98	1.10E-08	131.38	1.12E-09	**137.65**	**1.52E-09**
	75	135.43	2.64E-08	121.32	1.23E-07	127.95	4.12E-08	136.27	1.07E-09	129.01	1.24E-08	130.54	2.65E-08	**132.032**	**8.22E-08**

**Table 3:** SNR and SSIM performance for text messages of different lengths.

Audio file length (ms)	Text length	[3]		[22]		[23]		[24]		[25]		[26]		Proposed algorithm	
		SNR	SSIM	SNR	SSIM	SNR	SSIM	SNR	SSIM	SNR	SSIM	SNR	SSIM	SNR	SSIM
10	15	315.7	1	201.65	0.99	298.5	0.971	320.39	0.988	306.52	0.97	**310.12**	**1**	**317.04**	**1**
	30	250.74	0.999	198.43	0.989	237.34	0.965	281.41	0.985	251.39	0.961	**259.62**	**1**	**280.4**	**1**
	50	221.17	0.989	186.26	0.981	197.67	0.961	236.2	0.979	213.61	0.955	**227.76**	**0.998**	**234.01**	**0.999**
	75	213.89	0.988	180.54	0.979	151.46	0.959	228.53	0.975	189.22	0.951	**213.16**	**0.996**	**226.53**	**0.997**
7	15	292.2	0.9977	198.32	0.987	274.2	0.97	294.12	0.983	271.28	0.966	**291.83**	**0.999**	**292.01**	**1**
	30	226.06	0.9899	187.77	0.983	181.72	0.965	230.33	0.98	218.32	0.959	**230.52**	**0.997**	**228.04**	**0.998**
	50	200.63	0.9888	179.09	0.987	165.91	0.961	202.03	0.978	193.14	0.954	**206.87**	**0.996**	**200.08**	**0.998**
	75	189.16	0.9888	176.12	0.987	152.27	0.955	194.01	0.966	183.2	0.95	**192.8**	**0.996**	**190.89**	**0.996**
4	15	251.05	0.9977	181.56	0.976	197.65	0.96	261.16	0.97	251.61	0.959	**264.33**	**0.998**	**259.48**	**0.999**
	30	224.79	0.9899	172.23	0.971	177.33	0.959	227.31	0.965	206.36	0.957	**231.63**	**0.997**	**225.39**	**0.998**
	50	210.34	0.9875	164.65	0.963	169.56	0.957	214.15	0.96	201.62	0.953	**212.1**	**0.996**	**211.17**	**0.996**
	75	205.24	0.9899	152.33	0.961	150.03	0.951	208.03	0.959	189.13	0.949	**209.53**	**0.991**	**205.53**	**0.993**

In steganographic media (audio, image, or video), payload capacity is generally measured in bits. In theory, the embedding for each pixel (byte) is always lower than the pixel (bit) size. The size of the cover media (music, image, or video) and the method employed will both have an important effect on the Payload capacity, because, with a tiny cover size, but also needs a limited Payload capacity. As a result, the Bit per Byte (BPB) metric must also be included to assist in the presentation of the findings [56]. In other words, BPB enables the comparison of different steganography algorithms. Table 1 contains information on the database that was used.

Tables 2 and 3 show the quality measurement results before and after hiding text messages of different lengths (15, 23, 50, 75 characters) in speech signals of different lengths (10, 7, 4 ms), based on the PSNR, MSE, SNR and SSIM. The tables compare the methods described in [3, 22–26] with our proposed hiding algorithm. In our work, the SSIM values were close to +1, indicating a strong link between the original and stego-speech signals. The results for SNR and PSNR were higher than or very close to those of the alternative methods.

Tables 4 and 5 show a significant improvement when our method is used to embed messages in audio files. The difference is minimal, thus avoiding suspicion that stego-audio has been used. In addition, the noise reduction achieved by using SNR ensures greater confidentiality. The proposed algorithm can also provide more capacity whilst maintaining acceptable SNR results. The SNR values for our algorithm are slightly lower than for the scheme in [24], or very similar, which indicates that the stego-file was generated accurately and with good quality, since the LSB changes by only one bit. The SNR results from the proposed method are higher than the results for the scheme in reference [25], which also uses the LSB algorithm. This may be because the distinction of our work using LSB according to specific conditions depends on an XOR operation between the bits of the MSB to change the bit of one of the two bits of the LSB (not only the last bit in LSB).

**Table 4:** Comparison of SNR results.

Capacity (bytes)	[3]	[22]	[23]	[24]	[25]	[26]	Proposed algorithm
1	311.265	296.13	296.98	312.239	299.5404	310.101	310.021
10	301.388	268.59	271.891	304.321	285.1162	295.815	301.1232
100	222.92	179.81	183.342	229.771	197.329	209.919	227.2739
1000	194.941	159.26	166.701	199.827	171.5498	187.505	196.6159
10000	110.602	85.82	90.88	115.016	93.9801	108.022	111.0198

**Table 5:** Comparison of percentage differences between the cover and stego-audio.

Capacity (bytes)	[3]	[22]	[23]	[24]	[25]	[26]	Proposed algorithm
1	0.00911	0.00344	0.00171	0.000265	0.00451	0.00681	0.00035
10	0.0881	0.00264	0.00979	0.00114	0.03352	0.0667	0.00199
100	0.977	0.06631	0.0998	0.019	0.51211	0.7426	0.03251
1000	8.0897	3.0449	3.197	1.28	7.6212	7.9019	1.9771
10000	94.541	84.2206	86.0094	70.01	91.65414	92.911	71.7874

#### Perceptual evaluation of speech quality

We use the PESQ value here as a score to measure the usability of speech. We can determine the extent of carrier speech distortion by comparing the PESQ values of the original and reconstructed speech [57]. The value of this metric ranges from one to 4.5, where a score of one indicates that the cover and stego-audio files are not qualitatively equivalent, and a score of 4.5 means that they are. The threshold for acceptability for the PESQ is 3.8 [54, 58]. According to the values in Table 6, the results from the proposed stego-system indicate that a more stable embedding can be achieved than with the alternative techniques. The quality of the covert speech produced by this method is slightly lower than that of the original signal, indicating that the method proposed in this paper has a small effect on speech quality, as shown in Table 6.

**Table 6:** Comparison of MSE, SSIM, PESQ, MOS, CPU time (s), and memory (Kb).

Reference	MSE	SSIM	PESQ	MOS	Embedding CPU time (s)	Extraction CPU time (s)	Embedding memory allocation (Kb)	Extraction memory allocation (Kb)
[3]	2.49E-11	0.999	3.1019	4.8901	0.592	0.507	2533	7
[22]	9.21E-09	0.998	3.0002	4.2272	0.629	0.691	2901	8
[23]	8.50E-10	0.999	3.1018	4.361	0.481	0.49	2514	6
[23]	3.78E-12	1	3.8124	4.61	0.419	0.487	2744	6
[25]	3.99E-10	1	3.3232	4.6202	0.327	0.394	2538	5
[26]	2.90E-11	0.999	3.1008	4.9	0.304	0.218	2328	4
**Proposed scheme**	**4.00E-12**	**1**	**3.8026**	**4.644**	**0.191**	**0.108**	**1026**	**2**

#### Mean opinion score

In view of its availability, the MOS was used in this speech signal test. However, the International Telecommunications Union (ITU-T) [26] has presented a run system for subjective speech quality measurement. The MOS was used to evaluate the stego-and original signals by asking 10 people to listen to the speech, who then reported differences in the quality of the stego-signal and the original signal [26]. The average of these dissimilarity reports for speech MOS was then computed as shown in Table 6.

*Remark 4*. The proposed method outperforms the previous methods (Table 6) in terms of MSE, PESQ, and MOS, due to the use of a conditional LSB method that hides one bit in each sample of the cover speech signal, thus reducing the perceived percentage change in it.

### Time requirements and storage space complexity

The embedding time, extraction time, and memory are crucial factors affecting real-time applications and smart devices. In Table 6, the various steganography techniques are compared in terms of their storage and processing times. The proposed method has low time consumption and small storage requirements for hiding and extraction, due to its use of a lightweight hyperchaotic map based on the QM and the Hénon map. We also used a low-complexity LSB algorithm and a simple XOR operation at the hiding stage.

We note that the embedding and extraction processes described in [3, 22, 23], take a relatively long time compared to the proposed method, as the k-means technique is implemented in [3] and the one-time pad procedure is used to determine the hidden data locations in the audio signal. The genetic algorithm is used in [22] and the 4-wrap length technique in [23]. The embedding and extraction of secret messages in [24] take longer than in our scheme since they use DCT and SVD, which are time-consuming. The authors of [25] used the LSB algorithm and the one-time pad for embedding, which add extra time to the embedding and extraction processes. They also used the logistic map to generate the locations for hiding data in an audio signal. This introduces a weakness to its use as a type of chaotic map in its raw form without adding any embedding complexity, while the case is in our method, which relied on randomly generating hiding locations based on a hyperchaotic generator. We also note that the embedding and extraction times are higher than in our proposed method due to the need to generate two independent types of chaotic keys: one at the encryption stage, and the other at the embedding stage. Finally, we compare our work with the scheme in [26], and note the high execution time of latter scheme arises from the use of high-cost LPA, DWT, and SVD techniques. The “profile (‘-memory’, ‘on’)” function in MATLAB was used to compute the time and CPU memory for each steganography mechanism in this paper.

#### Robustness

Robustness is a measure of the resistance of secret messages to elimination or corruption, either intentionally or unintentionally, using various types of digital signal processing. Our experiment examined the BER for the original secret message and the extracted secret message in order to assess the robustness of our method [24]. BER is defined in Eq 8 as follows [24, 32]:

$$\begin{array}{c} BER=\frac{B_{err}}{M}\times 100\% \end{array}$$ (8)

Where BER is the number of erroneous bits, and M is the total number of bits.

In these experiments, we attacked the stego-speech signal separately using several typical signal processing attacks, as summarized in Table 7 [24, 26, 59, 60].

**Table 7:** Attack types [24, 26, 59, 60].

Attack	Explanation
Amplitude scaling	The amplitudes of the stego-speech are rescaled by ±30%
Re-sampling attack	16 kHz upsampling is applied to the stego-speech, followed by 8 kHz downsampling
Low-pass filtering (LPF)	The stego-speech is filtered with a low-pass filter, which has a cut-off frequency of 3.5 kHz
High-pass filtering (HPF)	The stego-speech is filtered with a high-pass filter, which has a cut-off frequency of 500 Hz
MP3 compression	Compression is applied to the stego-speech signal in MPEG-1 Layer III, with bit rates 128 and 96 kbps
Noise addition	Random noise is added to the stego-speech signals, where the SNR is 30 dB
AWGN	To imitate ecological error, a white Gaussian noise is applied to the signal.

As shown in Table 8, the proposed steganography technique has good robustness, as the BER is close to zero (owing to fewer errors between the original and extracted watermarks) [55].

**Table 8:** Comparison of the robustness (BER % and NCC) of different steganography techniques.

Attack	Method in [3]	Method in [22]	Method in [23]	Method in [24]	Method in [25]	Method in [26]	Method in [60]		Proposed method	
	BER %	BER %	BER %	BER %	BER %	BER %	BER %	NCC	BER %	NCC
Amplitude (0.7)	0.2211	0.4552	0.4191	0	0.3929	0.3802	0	1	**0**	**1**
Amplitude (1.3)	0.2209	0.4491	0.4099	0	0.3881	0.3684	0	1	**0**	**1**
Re-sampling	0.1309	0.491	0.3031	0.0032	0.271	0.1145	0.32	0.99	**0.003**	**0.99**
LPF (3.5 kHz)	0.0191	0.2618	0.1001	0.003	0.0967	0.0913	0.15	1	**0.0026**	**0.99**
HPF (500 Hz)	0.3299	0.4099	0.3991	0.0056	0.3981	0.3533	-	-	**0.0048**	**0.99**
MP3(128 kbps)	0.008	0.0301	0.0266	0	0.0182	0.0095	0.76	0.99	**0.0003**	**1**
MP3(96kbps)	0.0092	0.0204	0.0101	0	0.0091	0.0095	1.1	0.99	**0**	**1**
Noise(30dB)	0.0377	0.3011	0.2054	0.1473	0.0203	0.0113	0	1	**0.0711**	**0.99**
AWGN(5db)	-	-	-	-	-	0.1488	-	-	**0.0931**	**0.99**

Normalized cross-correlation (NCC) is an effective method for measuring the relationships between two processes, which may be utilized to determine how close the cover signal and the stego signal are, even after embedding data. The below equation is used to compute the NCC [55]:

$$\begin{array}{c} NCC\left(S,S^{\prime }\right)=\frac{\sum_{w=1}^{z}S\left(w\right)S^{\prime }\left(w\right)}{\sqrt{\sum_{w=1}^{z}S{\left(w\right)}^{2}}\sqrt{\sum_{w=1}^{z}S^{\prime }{\left(w\right)}^{2}}} \end{array}$$ (9)

where *S* and *S*′ are the raw and restored hidden messages, and *z* is the total amount of samples in each one.

The cover and stego audios are indistinguishable when tested with the conventional PESQ and MOS. It is clearly in Table 6, which demonstrates that our approach is protected from many attacks described in Table 7 and provides (as appears in Table 8) a perfect BER value close to zero and NCC score near +1, indicating that every one of the secret messages is fully recovered.

To continue analyzing the inaudibility tests, we look at the general MSE and SSIM results obtained by current studies in Table 6, which show that our strategy was effective and better than similar methods. In fact, the MSE values for the proposed technique are lower than in studies [24, 25], with our algorithm reaching 3.9927e-10 MSE rather than 3.9994e-12 and 3.7812e-12 MSE in studies [24, 25], respectively. Also, the values of SSIM compared to the two studies in [24, 25] achieved the value of 1.

*Remark 5*. The lower values in Table 8 for our technique compared to the other methods indicate the robustness of the proposed method. This is due to the suggested method’s resilience is strengthened by its reliance on hiding in specific eight bits ranging from 17 to 24 bits (selected experimentally) inside the original sixty-four bits of every block inside the cover speech signal. Although the nature of the chaos properties, which include the mixing property, quasi-randomness, and excellent autocorrelation properties. The scrambling algorithm is applied to the values of the text message in its binary form to scramble it in a complicated way. This is achieved through the use of two low-cost and fast mechanisms, the odd-even index and forward-backward reading, which are repeated at two levels: once on the entire text message in its binary form, and again after dividing it into three parts to add to the difficulty of retrieving it by hackers. Furthermore, the lightweight XOR operation used with the conditional LSB method makes the process more efficient and safer, and is more likely to prevent attackers from recovering hidden messages.

## Section 5: Conclusion

The fundamental goal of the proposed system, which involves hiding textual data in a .wav voice signal for communication over the web, is to provide an efficient, lightweight, and safe algorithm. We have achieved this by using complex strategies that are difficult to break. This study has proposes a random method of determining the location for the insertion of a text file before it is hidden in the speech signal using a conditional LSB algorithm. Furthermore, the proposed algorithm for scrambling input text in its binary representation makes text recovery more complex. Our method yields perfect results, with a concealed text that cannot be identified or restored. The results show that the size of the speech file stays unchanged after hiding the message. The same audio signal file was used to incorporate several text files with varying sizes of content, and it was found that the cover file was not lost or altered, and the results were satisfactory (as indicated in Figs 3 and 4). The proposed method was also evaluated using MSE, SNR, PSNR, SSIM, PESQ, and MOS imperceptibility tests (as shown in Tables 1–6), and the results showed that our approach outperformed existing related schemes based on these metrics. When an audio file titled “speech1.wav” with a payload capacity of 6.5 percent is applied, the suggested strategy enhances the PSNR and SNR, which reach 91.317 and 161.493, respectively. Also, the MSE value was reduced to 1.642e-10 for the same speech file with the same capacity, which reflects good results compared to the references [3, 24, 25] shown in Table 1. The execution time of our model is short (Embedding CPU time and Extraction CPU time equals 0.191, and 0.108 respectively as show in in Table 6), as it uses three lightweight algorithms: the first is based on lightweight chaotic systems, namely the QM and the Hénon map, which are used in the generated hyperchaotic map; the second is a simple, safe scrambling algorithm that depends on a technical scrambling strategy; and the third is a conditional LSB algorithm that uses a low-complexity XOR operation. The time required for the execution of our method satisfies the requirements for real-time communication and application. All these findings offer strong support for the implementation of the proposed method on smart devices. Future research will focus on improving the concealment and real-time capabilities of this technique, and on applying it to other kinds of data such as video and images.
